# Novel binding pocket descriptors based on DrugScore potential fields encoded by 3D Zernike descriptors

**DOI:** 10.1186/1758-2946-4-S1-P28

**Published:** 2012-05-01

**Authors:** Britta Nisius, Fan Sha, Holger Gohlke

**Affiliations:** 1 Department of Mathematics and Natural Sciences, Institute of Pharmaceutical and Medicinal Chemistry, Heinrich-Heine University, Universitätsstraße 1, 40225 Düsseldorf, Germany

## 

Proteins interact with other molecules, e.g. ligands or other proteins, in specific binding sites. Key factors for these interactions are the shape, size, and buriedness of the binding site, as well as its physicochemical composition. Since all these properties usually significantly vary among different proteins, up to now there is no standard definition what constitutes a binding site [1]. Thus, novel pocket descriptors allowing an in-depth characterization of binding sites are highly desired.

Hence, we developed novel binding pocket descriptors based on 3D molecular interaction fields. The binding pocket of a protein is characterized using the distance dependent, knowledge-based pair potentials of the DrugScore scoring function [2] in combination with multiple ligand atom probes. To allow an efficient comparison of the resulting potential fields, the 3D grids are encoded using 3D Zernike descriptors.

The 3D Zernike polynomials *Z_nlm_* are orthonormal basis functions on the unit sphere. Thus, any 3D object can be represented as:

using a 3D Zernike function expansion. We utilized the resulting function expansion coefficients *c_nlm_*, i.e. the 3D Zernike moments, to describe the 3D molecular potential fields characterizing a protein's binding pocket.

The resulting descriptors are invariant under rotation, scaling, and translation and enable a fast comparison and an efficient characterization of protein binding pockets. Thus, these novel pocket descriptors can be used to predict target druggability or to calculate similarities between binding pockets, e.g. to predict potential off-targets or to perform protein function de-orphanization.
